# IMPACT OF TOTAL KNEE ARTHROPLASTY ON PHYSICAL ACTIVITY

**DOI:** 10.1093/geroni/igac059.062

**Published:** 2022-12-20

**Authors:** Annalisa Na, Olivia Rosenstein, Tony Chao, Chad Davenport, Karen Chapman, Ronald Lindsey, Zbigniew Gugala

**Affiliations:** Drexel University, Philadelphia, Pennsylvania, United States; Drexel University, Philadelphia, Pennsylvania, United States; University Of Texas Medical Branch, Galveston, Texas, United States; University of Texas Medical Branch, Galveston, Texas, United States; University of Texas Medical Branch at Galveston, Galveston, Texas, United States; University of Texas Medical Branch at Galveston, Galveston, Texas, United States; University of Texas Medical Branch, Galveston, Texas, United States

## Abstract

Knee osteoarthritis (OA) pain often challenges the physical activity necessary for managing life-threatening chronic diseases. Standard severe knee OA treatment of total knee arthroplasty (TKA) and physical therapy (PT) is effective at improving pain and function but whether such benefits translate to physical activity is unclear. We enrolled 22 participants with severe knee OA scheduled for a TKA (Age,mean±SD=69.0±5.8y, female=63.6%) and assessed pain (i.e.,Numeric Pain Rating), physical function (i.e.,Knee Osteoarthritis Outcome Score), and physical activity (i.e.,activity monitors and journals for 7-days) before and 1-month, 3-month, and 6-month after TKA. Using paired-wise ANOVA, pre-to-postTKA pain and function improved but not physical activity. Using regression analyses, outpatient PT sessions during first two months post-TKA were positively related to 3- and 6-month physical activity (r=0.51-070, P=0.003-0.029). Standard TKA and PT for severe OA improved pain and function but not physical activity. However, early post-TKA PT and physical activity relationships are promising, warranting exploration.

